# The effect of pectin-supplemented enteral nutrition in mechanically ventilated patients receiving gastric feeding: protocol of a multicenter, open-label, randomized controlled trial (the PROMOTE trial)

**DOI:** 10.3389/fmed.2026.1816835

**Published:** 2026-06-23

**Authors:** Zirui Liu, Yizhen Xu, Yuan Ding, Lin Gao, Ruixin Bai, Cheng Lv, Qian Li, Man Li, Yingqiao Bai, Xiang Li, Wei Qin, Wangye Wu, Wencheng Yang, Zhengquan Wang, Jun Lu, Yuxiu Liu, Lu Ke, Feng Zheng, Baiqiang Li, Weiqin Li

**Affiliations:** 1 Department of Critical Care Medicine, Jinling Hospital, Affiliated Hospital of Medical School, Nanjing University, Nanjing, Jiangsu, China; 2 Research Institute of Critical Care Medicine and Emergency Rescue at Nanjing University, Nanjing, Jiangsu, China; 3 Jinling Clinical Medical College, Nanjing Medical University, Nanjing, Jiangsu, China; 4 Emergency and Critical Care Center, Department of Emergency Medicine, Zhejiang Provincial People’s Hospital (Affiliated People’s Hospital, Hangzhou Medical College), Hangzhou, Zhejiang, China; 5 Department of Critical Care Medicine, Zhejiang University School of Medicine Sir Run Run Shaw Hospital, Hangzhou, Zhejiang, China; 6 Department of Critical Care Medicine, Liuzhou People’s Hospital, Liuzhou, Guangxi, China; 7 Department of Emergency Medicine, Hunan Provincial people’s Hospital, Changsha, Hunan, China; 8 Department of Critical Care Medicine, Lianyungang Municipal Oriental Hospital, Lianyungang, Jiangsu, China; 9 Intensive Care Unit, The Second Affiliated Hospital of Guilin Medical University, Guilin, Guangxi, China; 10 Department of Emergency Medicine, The Affiliated Yangming Hospital of Ningbo University, Yuyao, Zhejiang, China; 11 Department of Intensive Care Medicine, Affiliated Hospital of Nanjing University of Chinese Medicine, Jiangsu Hospital of Chinese Medicine, Nanjing, Jiangsu, China; 12 Department of Biostatistics, School of Public Health, Nanjing Medical University, Nanjing, Jiangsu, China; 13 National Institute of Healthcare Data Science, Nanjing University, Nanjing, Jiangsu, China; 14 Department of Critical Care Medicine, Changshu Hospital Affiliated to Soochow University (Changshu No.1 People’s Hospital), Changshu, Jiangsu, China

**Keywords:** critical illness, enteral nutrition, pectin, gastric feeding, feeding intolerance

## Abstract

**Introduction:**

Enteral nutrition (EN) is the preferred route of medical nutrition therapy for critically ill patients. However, enteral feeding intolerance remains a frequent challenge when implementing EN. Pectin, a water-soluble dietary fiber, has been shown to reduce diarrhea and improve feeding tolerance, but existing evidence remains inconsistent. The PROMOTE trial aims to evaluate whether EN supplemented with pectin, compared to usual care, improves EN delivery in critically ill patients.

**Methods:**

This is a multicenter, open-label, randomized, parallel-controlled trial, conducted in 10 centers across China. Newly admitted adult critically ill patients who received invasive mechanical ventilation for less than 72 h and had an indication for EN via gastric access will be enrolled. In addition to standard care, patients assigned to the intervention group will receive EN supplemented with 90 g of pectin per 500 mL of EN solution. The study intervention will be discontinued 7 days after randomization, upon discharge from the study site, upon death, or upon cessation of gastric feeding, whichever occurs first. The primary outcome is the ratio of EN energy intake to the prescribed target energy on Day 5 after randomization. Secondary outcomes include daily energy and protein intake, the incidence of enteral feeding intolerance, duration of ICU stay, duration of mechanical ventilation, duration of hospital stay, 28-day mortality, ICU-free, hospital-free and ventilation-free days to 28 days.

**Discussion:**

This study investigates whether pectin-supplemented EN enhances gastric feeding tolerance and clinical outcomes in critically ill patients. The finding will provide evidence on pectin’s efficacy in optimizing EN delivery and guide early gastric feeding strategies in critical care.

**Clinical trial registration:**

https://www.chictr.org.cn, identifier ChiCTR2300078477.

## 1 Introduction

Early initiation of enteral nutrition (EN) is critical for critically ill patients. Both ASPEN and ESPEN guidelines recommend starting EN with 48 h rather than delaying EN or initiating early parenteral nutrition (PN) (1, 2). Gastric delivery remains the standard initial route. However, EN frequently fails due to feeding intolerance, which affects approximately 20%–30% of critically ill patients and presents with elevated gastric residuals, nausea, vomiting, abdominal distension, or diarrhea (3, 4). Furthermore, this intolerance is associated with serious risks such as aspiration, inhalation pneumonia, and potentially fatal outcomes (5).

Current strategies for managing feeding intolerance are limited. Initial measures include prokinetic agents (e.g., erythromycin, metoclopramide) and formula adjustments, followed by post-pyloric feeding if needed (6, 7). If intolerance persists despite these interventions, PN is initiated. However, this stepwise approach is not always effective and can be time-consuming, potentially depriving patients of adequate EN and its associated benefits (7, 8). Feeding intolerance may be directly related to EN itself (9). For instance, the high osmolarity of EN formulations can contribute to diarrhea (10), while the low viscosity and high infusion rate may increase the risk of gastroesophageal reflux and subsequent aspiration (11).

Pectin, a gelatinous substance derived from the cell walls of fruits and plants, is a major component of water-soluble dietary fiber and possesses multiple biological activities. In the acidic gastric environment, pectin can interact with calcium ions (Ca^2+^) to form a semi-solid gel (12, 13). Increasing the viscosity of EN with pectin may therefore reduce diarrhea and reflux aspiration risk (14). A systematic review and meta-analysis showed that pectin-supplemented EN was associated with reduced diarrhea, fewer infectious complications, and a shorter length of stay (15). Notably, its effect was evident in patients not receiving proton pump inhibitors (PPIs) but absent in those on PPIs (16), likely because conventional pectin requires an acidic environment to gel. Low-esterified pectin, however, can form a stable hydrogel even under low-acid conditions, via a pH-independent Ca^2+^-mediated gelation mechanism (17), thereby increasing intragastric content cohesiveness and reducing retrograde flow and reflux-driven interruptions. This distinguishes our intervention from generic fiber supplementation and broadens its applicability to the general critically ill population.

Although pectin supplementation appears promising in reducing feeding intolerance and improving clinical outcomes, most existing evidence comes from observational studies or small, single-center randomized controlled trials (RCTs). To address this gap, we plan to conduct a multicenter RCT (PROMOTE trial). Our hypothesis is that EN supplemented with low-esterified pectin, a physiochemically more robust form of pectin, will improve feeding tolerance and enable greater total energy delivery compared with usual care in critically ill adults.

## 2 Methods and analysis

### 2.1 Study design, setting, and population

PROMOTE is a multicenter, open-label, randomized, parallel-controlled trial conducted by the Chinese Critical Care Nutrition Trials Group (CCCNTG). The trial will enroll 212 critically ill adult patients from ten intensive care units (ICUs) across China. Trial oversight will be provided by a Trial Management Committee (TMC), comprising the principal investigators from each participating center and key members of CCCNTG, which will be responsible for the overall conduct and coordination of the study. Recruitment started in February 2024 with competition for enrollment expected in 2026. The trial protocol was developed according to the Standard Protocol Items: Recommendations for Interventional Trials (SPIRIT) statement (18).

Patients aged ≥ 18 years and within 24 h of their index ICU admission will be screened for eligibility. Those requiring invasive mechanical ventilation and, therefore, unable to take oral nutrition, necessitating artificial nutritional support, will be eligible for inclusion. The complete inclusion and exclusion criteria are listed below:

#### 2.1.1 Inclusion criteria

1. Aged ≥ 18 years old;

2. Admitted to ICU < 24 h;

3. Receive invasive mechanical ventilation < 72 h, and the expected duration of continued treatment > 48 h;

4. Do not expect to feed orally for more than 3 days and plan to receive gastric feeding;

5. No operation plan within a week;

6. Informed consent form obtained from the patient or next of kin.

#### 2.1.2 Exclusion criteria

1. Patients who had started EN before admission to ICU;

2. Patients who have had a nasointestinal tube placed and are scheduled to receive enteral feeding;

3. Patients with contraindications to EN;

4. Patients receiving palliative care or expected to die within 48 h;

5. Patients known to be pregnant or currently breastfeeding;

6. The treating clinician believes a specific high-calorie (or low-calorie) nutritional formula is indicated.

### 2.2 Randomization and blinding

The randomization sequence will be generated by an independent statistician using SAS Version 9.4 with a fixed block size of 4, ensuring allocation in a 1:1 ratio. Randomization will be stratified by study site. Allocation concealment will be ensured through a secure, encrypted, password-protected central web-based randomization system.^1^

Blinding of participants and treating clinicians was not feasible because pectin supplementation changes the handling and administration characteristics of the enteral formula, making it distinguishable from standard care during routine clinical practice. No placebo will be provided to the control group. Nonetheless, outcome assessors and data analysts will remain blinded to group assignments to minimize assessment and analytic bias.

### 2.3 Intervention and comparator

#### 2.3.1 Intervention group

The intervention will consist of administering pectin-supplemented EN from randomization until the earliest of the following events: (1) discontinuation of gastric feeding, either due to successful transition to full oral intake or intolerance requiring a switch to post-pyloric feeding or PN; (2) ICU discharge; (3) death; or (4) 7 days after enrollment.

The pectin supplement (Yaoshun^®^ citrus pectin, Qirui medicine, China) consists of low-esterified citrus pectin with a degree of esterification of 5%–15% and is formulated to maintain relatively low viscosity during tube administration. In the presence of calcium ions contained in the enteral formula, the pectin can undergo calcium-mediated gelation, forming a semi-solid gel and increasing intragastric viscosity, which may help reduce retrograde flow and gastroesophageal reflux. The supplement will be administered at a total daily dose of 90 g per 500 mL of enteral formula, delivered as divided 45 g boluses via gastric tube. One bolus will be administered immediately before initiation of EN, with subsequent boluses given after every 250 mL of formula infusion. To minimize the risk of tube occlusion, the feeding tube will be flushed with 20 mL of warm water before and after each pectin administration.

#### 2.3.2 Control group

The comparator will be usual nutritional care, delivered and managed in accordance with the CCCNTG feeding protocol^2^ based on international guidelines (19).

#### 2.3.3 ICU procedures common to both arms

Once randomized, the treating clinical team will determine the target infusion rate (mL/hour) for continuous EN to meet the energy requirement. Selection of the EN formula, estimation of protein needs, and blood glucose management will follow the CCCNTG feeding protocol or the standard procedures of each participating site.

Feeding intolerance will be managed according to the CCCNTG feeding protocol. Initial strategies include the use of prokinetic agents (e.g., erythromycin, metoclopramide) and adjustments to the EN formula. For patients with persistent gastric intolerance despite prokinetic therapy, postpyloric feeding will be implemented. If intolerance continues even after postpyloric feeding is attempted, PN will be commenced.

All patients will receive routine clinical care as determined by the local ICU team, including monitoring of vital signs, analgesia and sedation, fluid therapy, mechanical ventilation, renal replacement therapy, and vasoactive support. All cointerventions will be undertaken at the discretion of the treating clinicians and documented in the medical record.

### 2.4 Study outcomes and their measures

#### 2.4.1 Primary outcome

The primary outcome of this study is the ratio of EN energy intake to the prescribed target energy on Day 5 after randomization. Patients who died before Day 5 will not be included in the analysis of the primary outcome. Patients who were discharged from ICU before Day5 will be followed up after ICU discharge.

Target energy requirements will be estimated using a simple weight-based equation of 25 kcal/kg of body weight. Body weight used for calculating energy requirements will follow the recommendations of the 2023 ESPEN guideline (2): actual body weight will be used if the body mass index (BMI) is ≤25. For patients with BMI > 25, an adjusted body weight will be applied, defined as:

Adjusted body weight = Ideal body weight + 25% × (actual body weight–ideal body weight). Ideal body weight (kg) will be determined using the formula: 0.9 × height (cm)–100 for males, and 0.9 × height (cm)–106 for females.

#### 2.4.2 Secondary outcomes

Part I: Clinical outcomes

1. Duration of ICU stay.

2. Duration of mechanical ventilation.

3. Duration of hospital stay.

4. 28-day mortality.

5. ICU-free to 28 days.

6. Hospital-free days to 28 days.

7. Ventilation-free days to 28 days.

8. Incidence of new-onset organ failure within 7 days after enrollment.

9. New receipt of organ support within the first 7 days.

10. Number of organ support–free days within the first 7 days.

11. Daily Sequential Organ Failure Assessment (SOFA) scores recorded over the first 7 days.

Part II: Nutritional outcomes

1. Time to initiation of EN.

2. Average daily energy intake within the first 7 days.

3. Average daily protein intake within the first 7 days.

4. Daily Bristol stool score within the first 7 days.

5. Daily Feeding Intolerance Score (FIS) (20) within the first 7 days (Supplementary Table 1).

6. Daily Gastrointestinal Dysfunction Score (GIDS) (21) within the first 7 days (Supplementary Table 2).

7. Proportion of patients who transition to postpyloric feeding within the first 7 days.

8. Proportion of receiving PN within the first 7 days.

9. Caloric adequacy on Days 3, 5, and 7, defined as achieving at least 80% of the estimated energy target.

10. Protein adequacy on Days 3, 5, and 7, defined as achieving at least 70% of a target of 1.3 g/kg/day.

Part III: Biochemical outcomes

Daily serum albumin, prealbumin, C-reactive protein (CRP), and blood urea nitrogen (BUN) during the first 7 days after enrollment.

Outcome definitions are as follows. *New-onset organ failure* is defined as failure arising in an organ system that was functionally intact at randomization, corresponding to an increase of ≥2 points in the SOFA sub-score for the respiratory, circulatory, or renal system relative to baseline. SOFA scores are assessed daily for the first 7 days following randomization. *New receipt of organ support therapy* refers to the initiation of mechanical ventilation, renal replacement therapy, or vasoactive agents that were not being used at enrollment. *Daily energy and protein intake* will be calculated based on EN, PN, and quantifiable oral nutritional supplements, with non-quantifiable oral intake excluded.

#### 2.4.3 Duration of treatment and follow-up

The study intervention will be discontinued 7 days after randomization, discharge from the study ICU, death or cessation of gastric feeding, whichever comes first. All randomized patients will be followed for 28 days after randomization, unless death occurs earlier. For patients who remain hospitalized on study day 28, follow-up will be censored at day 28, and all outcomes will be recorded at that time point.

#### 2.4.4 Trial termination and intervention discontinuation

Participants may withdraw from the study at any time and for any reason, without any impact on their current or future clinical care provided by the investigators or treating clinicians. Investigators are required to document the reason(s) for withdrawal. The extent of withdrawal (e.g., discontinuation of study intervention, termination of active study participation, or cessation of all study-related contact) will be recorded in the electronic Case Report Form (eCRF). Post-randomization follow-up of withdrawn participants will be pursued to the extent permitted by participant consent. If consent is provided, follow-up assessments may be conducted by telephone.

The study intervention (pectin) will be immediately discontinued under any of the following circumstances:

1. Occurrence of a serious adverse event (SAE) or a concurrent medical condition for which continuation of pectin is considered not to be in the participant’s best interest, as judged by the investigator.

2. Withdrawal of informed consent by the participant.

The reason(s) and date(s) for intervention discontinuation or study withdrawal will be documented in the eCRF. If pectin is discontinued due to an SAE, safety monitoring will continue until the event has resolved or stabilized, or until it is determined to be unrelated to the study intervention. All relevant adverse events will be formally recorded in accordance with study procedures.

### 2.5 Data collection and management

Data will be collected and stored using a secure, web-based electronic data capture system (Unimed Science Inc., Wuxi, China). At each participating center, the principal investigator will be responsible for oversight of patient enrollment and data entry. Data entry will be performed exclusively by the principal investigator or by up to two designated research staff members authorized by the principal investigator.

Standardized training in data entry and database use will be provided by the database vendor in collaboration with the CCCNTG coordinating center. All data required to characterize baseline patient characteristics (Table 1), study and control interventions, potential confounding co-interventions, and study outcomes will be systematically and prospectively collected in accordance with the study protocol. Table 2 shows the schedule of enrolment, interventions, and main data collected.

**TABLE 1:** Baseline characteristics of patients by treatment group.

Characteristics	Pectin group (*n* = xx)	Control group (*n* = xx)
Age, years		
Male gender, no. (%)		
BMI, kg/m^2^		
Admission diagnosis, no. (%)		
Respiratory		
Cardiovascular		
Gastrointestinal		
Neurological		
Sepsis		
Trauma		
Others		
ICU source of admission, no. (%)		
Emergency		
Medical		
Surgical		
Others		
Comorbidities, no. (%)		
Hypertension		
Coronary disease		
Diabetes		
Chronic respiratory diseases		
Stroke		
Gastrointestinal disease		
Malignant tumor		
Others		
Interval from ICU admission to randomization, days		
APACHE II score		
SOFA score		
mNUTRIC score		
Presence of organ failure at baseline, no. (%)		
Respiratory		
Renal		
Cardiovascular		
Organ support at baseline, no. (%)		
Renal replacement therapy		
Vasoactive drugs		
Serum albumin, g/L		
Serum creatinine, μmol/L		
BUN, mmol/L		
CRP, mg/L		
PPIs use before randomization, no. (%)		

BMI, body mass index; ICU, intensive care unit; APACHE II, Acute Physiology and Chronic Health Evaluation II; SOFA, Sequential Organ Failure Assessment; mNUTRIC, modified Nutrition Risk in the Critically Ill; BUN, blood urea nitrogen; CRP, C-reactive protein; PPI, proton pump inhibitor.

**TABLE 2:** Schedule of enrolment, intervention, and data collection.

	Study period									
Time point	Screening	Randomiza-tion	After randomization/day							End
		0	1	2	3	4	5	6	7	28
Enrollment										
Eligibility screen	×	–	–	–	–	–	–	–	–	–
Informed consent	×	–	–	–	–	–	–	–	–	–
Allocation	–	×	–	–	–	–	–	–	–	–
Intervention										
Intervention group	–	×	×	×	×	×	×	×	×	–
Control group	–	×	×	×	×	×	×	×	×	–
Assessments										
Baseline characteristics	×	×	–	–	–	–	–	–	–	–
Laboratory parameters	–	–	×	×	×	×	×	×	×	–
APACHE II score	–	–	×	×	×	×	×	×	×	–
SOFA score	–	–	×	×	×	×	×	×	×	–
mNUTRIC score	–	–	×	×	×	×	×	×	×	–
Feeding Intolerance Score (FIS)	–	–	×	×	×	×	×	×	×	–
Gastrointestinal Dysfunction Score (GIDS)	–	–	×	×	×	×	×	×	×	–
Bristol stool score	–	–	×	×	×	×	×	×	×	–
Daily energy intake	–	–	×	×	×	×	×	×	×	–
Daily protein intake	–	–	×	×	×	×	×	×	×	–
Caloric adequacy	–	–	–	–	×	–	×	–	×	–
Protein adequacy	–	–	–	–	×	–	×	–	×	–
New-onset organ failure	–	–	×	×	×	×	×	×	×	–
Receipt of organ support	–	–	×	×	×	×	×	×	×	–
Clinical outcomes	–	–	–	–	–	–	–	–	–	×
Adverse events	–	–	×	×	×	×	×	×	×	×

APACHE II, Acute Physiology and Chronic Health Evaluation II; SOFA, Sequential Organ Failure Assessment; mNUTRIC, modified Nutrition Risk in the Critically Ill.

### 2.6 Sample size estimation

Based on a previous nationwide multicenter trial (the NEED trial) conducted by the CCCNTG (19), among patients receiving invasive mechanical ventilation, the mean (standard deviation) ratio of EN energy intake to the prescribed target energy on Day 5 was 62.9% (31.1%) in the control group. Assuming a minimal clinically meaningful relative increase of 20% in the intervention group, with 80% power, a two-sided alpha of 0.05, and a 1:1 allocation ratio, the required sample size is 96 participants per arm (PASS ver.11.0; NCSS, LLC). To account for potential attrition, including withdrawal or dropped, estimated at approximately 10%, the final sample size was increased to 212 participants (106 per group).

### 2.7 Data analysis

Reporting and presentation of trial results will adhere to the Consolidated Standards of Reporting Trials (CONSORT) guidelines (22). The primary analysis will be conducted according to the intention-to-treat (ITT) principle, with secondary supportive analyses performed in the per-protocol (PP) population and safety analyses in the safety population.

Analysis populations are defined as follows. The ITT population comprises all randomized participants. The PP population comprises participants who completed the intervention in accordance with the study protocol, excluding those with major protocol deviations, defined as: (a) enrollment of participants subsequently confirmed ineligible; (b) receipt of <80% of the planned pectin dose. All deviations will be prospectively recorded in a Protocol Deviation Log in the eCRF and reviewed by the TMC at each monitoring visit. The safety population comprises all participants who received at least one dose of the study intervention.

Primary outcome analysis: Patients who die before Day 5 are excluded from the primary analysis as a pre-specified competing event. A sensitivity analysis will assign these patients an EN delivery ratio of 0% (worst-case assumption), reported alongside the primary complete-case analysis. Administratively missing primary outcome data are anticipated to be <5% based on prior NEED trial experience (19) and will be handled by complete-case analysis with transparent reporting of the missing data rate. The number of deaths before Day 5 in each arm will be reported in the CONSORT flow diagram and primary analysis table to ensure full ITT accounting.

Time-to-event analyses: A competing risks framework using the Fine-Gray subdistribution hazard model, with death as the competing event, will be applied to time-to-event outcomes including time to achieve ≥ 80% of the prescribed EN target, time to ICU discharge, and time to hospital discharge, etc. Kaplan-Meier curves for 28-day mortality will be presented for both groups with log-rank comparison.

Statistical methods: Normality of continuous variables will be assessed using the Shapiro-Wilk test. Normally distributed continuous variables will be summarized as mean ± standard deviation and compared using Student’s *t*-test; non-normally distributed variables will be summarized as median (interquartile range, IQR) and compared using the Mann-Whitney U test. Categorical variables will be summarized as counts and percentages and compared using Pearson’s chi-square test. Effect estimates with 95% confidence intervals (CIs) will be reported throughout. No adjustment for multiple comparisons will be applied; all secondary outcome analyses are considered exploratory and hypothesis-generating. For secondary outcomes, complete-case analysis will be used, with missing data rates reported for each outcome. A two-sided *P*-value < 0.05 will be considered statistically significant.

Subgroup analyses: Pre-specified subgroup analyses will be conducted according to the following baseline characteristics: age (>60 vs. ≤59 years), BMI (>25 vs. ≤25 kg/m^2^), SOFA score (≥9 vs. <9), and ICU admission type (emergency, surgical, medical, or other).

### 2.8 Data and safety monitoring

An independent Data and Safety Monitoring Board (DSMB), comprising experts in clinical trials, biostatistics, and intensive care medicine, will be established to safeguard participant interests and monitor intervention safety throughout the trial. No formal interim efficacy analyses are planned. This decision was made at the protocol design stage and is consistent with the pragmatic nature of this trial. The DSMB will conduct safety reviews after every 50 participants complete the intervention, focusing on cumulative incidence of pre-specified SAEs, including intestinal ischemia, severe abdominal distension, and aspiration pneumonia and etc. The DSMB may recommend early termination on safety grounds at any time and the TMC will act on such recommendations within 48 h.

### 2.9 Adverse events

Adverse events (AEs) are defined as any unfavorable medical occurrence in a study participant receiving the investigational intervention, regardless of its causal relationship to the intervention, in accordance with the National Cancer Institute Common Terminology Criteria for Adverse Events (NCI-CTCAE v5.0) (23).

Given the severity of underlying illness in critically ill patients, abnormalities in laboratory values, signs, or symptoms are common and will not automatically be classified as AEs unless they require clinically significant intervention or are considered clinically relevant at the investigator’s discretion. The underlying condition or diagnosis should be documented whenever possible (e.g., “acute kidney injury” rather than “hyperkalemia”).

AE assessment follows a two-level framework: (1) all-cause AE grading using NCI-CTCAE v5.0; and (2) the following pectin-specific adverse events of interest, which will be actively monitored throughout the intervention period:

a. Abdominal distension: clinical diagnosis based on physical examination and/or intra-abdominal pressure (IAP) ≥ 12 mmHg on routine bladder pressure measurement, corresponding to Grade I intra-abdominal hypertension (IAH) per the World Society of the Abdominal Compartment Syndrome (WSACS) consensus (24).

b. Enteral tube obstruction: inability to aspirate gastric contents or flush the tube with 20 mL warm water, necessitating tube replacement or unplanned cessation of enteral feeding.

c. Ileus: absence of bowel sounds on ≥3 consecutive assessments combined with cessation of stool passage and abdominal distension in the absence of mechanical obstruction, requiring clinical intervention.

d. Intestinal ischemia: radiologically or surgically confirmed bowel ischemia, or strong clinical suspicion based on rising lactate and unexplained hemodynamic deterioration, prompting urgent computed tomography (CT) evaluation. Classified as SAE with mandatory immediate DSMB notification.

e. Aspiration pneumonia: new radiological infiltrates consistent with aspiration pneumonia occurring within 24 h of pectin administration and judged by the investigator as potentially related to altered gastric viscosity.

Causality will be assessed by the site investigator using the WHO five-category classification (Certain/Probable/Possible/Unlikely/Unrelated); events judged Possible or above are included in the safety analysis. A SAE is defined as any event resulting in death, life-threatening condition, prolonged hospitalization, or persistent significant disability. Non-serious AEs will be documented in the eCRF within 24 h of detection. SAEs will be reported to the ethics committee and DSMB in accordance with regulatory requirements.

## 3 Discussion

Enteral nutrition is the preferred route of medical nutrition therapy for critically ill patients (25). However, enteral feeding intolerance remains a frequent challenge in daily clinical practice (4, 26). Feeding intolerance often results in suboptimal nutrient delivery and gastrointestinal (GI) disturbances, and has been associated with prolonged mechanical ventilation, extended ICU and hospital length of stay, and increased mortality (4, 27).

To improve GI tolerance in critically ill patients, enteral formulas supplemented with dietary fiber have been developed and increasingly used (28). Current guidelines provide inconsistent recommendations regarding fiber supplementation in the ICU setting. The ASPEN guideline suggests considering the routine use of fermentable soluble dietary fiber in stable medical and surgical ICU patients (1), whereas the ESPEN guideline does not specifically address dietary fiber use in critical illness (2). A recent systematic review and meta-analysis including 20 RCTs demonstrated very low-certainty evidence suggesting potential clinical benefits of fiber-supplemented EN (29). Furthermore, trial sequential analysis indicated that the cumulative information size remains insufficient, highlighting the need for additional well-designed trials to clarify its efficacy.

Pectin, a fermentable soluble dietary fiber, has been proposed as a promising option to improve feeding tolerance and control diarrhea in critically ill patients. Several biological mechanisms may underlie these effects. Upon exposure to gastric acid, pectin undergoes gel formation, which may slow gastric emptying and prevent rapid intestinal transit (14). In the colon, the water-soluble fibers of pectin exhibit high water-binding capacity, potentially increasing stool consistency and attenuating excessive propulsive colonic contractions (30). In addition, pectin is fermented by colonic microbiota into short-chain fatty acids (SCFAs) (31), which serve as the primary energy source for colonocytes. SCFAs promote mucosal repair and enhance intestinal barrier integrity, thereby reducing bacterial translocation and potentially improving GI function and feeding tolerance (32, 33). Last, pectin may form a protective physiological barrier along the intestinal epithelium, limiting pathogen adherence and contributing to the maintenance of intestinal microbial homeostasis (34).

Diarrhea represents one of the most common manifestations of EN intolerance in the critically ill, with reported rates of 10%–70% among ICU patients receiving EN, reflecting genuine variability across populations, feeding routes, and definitions (9, 15). Given the multiple mechanisms by which pectin may attenuate diarrhea, including increased stool consistency, modulation of colonic transit, and enhancement of intestinal barrier integrity, diarrhea incidence is therefore included as a pre-specified secondary outcome to provide exploratory evidence on pectin’s antidiarrheal effects in this population.

Several limitations should be acknowledged. First, no placebo was provided to patients assigned to the control group, which may introduce performance bias. A matched placebo was not feasible, as low-esterified pectin rapidly forms a visible and palpable gel via Ca^2+^-mediated crosslinking when mixed with enteral formula, and no biologically inert alternative can currently replicate this transformation. To address this potential bias, treating physicians responsible for nutritional therapy were independent of the study investigators and remained blinded to treatment allocation. Furthermore, both groups were managed under the same standardized CCCNTG feeding protocol to ensure uniform nutritional management, and all outcome assessments and statistical analyses were conducted by personnel blinded to treatment allocation.

Second, the control group receives usual nutritional care without a mandated fiber-free formula, which may introduce confounding if fiber-containing formulas are used. Given the limited use of fermentable soluble fiber in standard ICU enteral formulas across participating centers, the magnitude of this confounding effect is expected to be modest.

Third, the viscosity-enhancing properties of low-esterified pectin may compromise the reliability of gastric residual volume (GRV) measurement in the intervention group, as the viscoelastic gel formed in the gastric lumen can resist aspiration through a nasogastric tube, leading to systematic underestimation of true residual content. As our protocol does not mandate GRV-based EN management, consistent with current evidence and guideline recommendations, EN tolerance is assessed using functional clinical endpoints. The relationship between pectin-induced viscosity and gastric emptying in critically ill patients warrants further investigation.

Fourth, given the 7-day intervention period, the trial is not powered to detect differences in longer-term clinical outcomes; secondary outcomes should therefore be interpreted as exploratory and hypothesis-generating.

In summary, this multicenter RCT aims to address an important gap in the current evidence regarding dietary fiber supplementation in critically ill patients. By rigorously evaluating the effects of low-esterified pectin on feeding tolerance and EN delivery, the findings have the potential to advance our understanding of the role of dietary fiber in EN for critically ill adults and inform the design of future confirmatory trials.

## Footnotes

1
https://capctg.medbit.cn/en/2024/01/08/845/

2
https://capctg.medbit.cn/
