# Supplementary material for: The effect of pectin-supplemented enteral nutrition in mechanically ventilated patients receiving gastric feeding: protocol of a multicenter, open-label, randomized controlled trial (the PROMOTE trial)
Source: Front Med (Lausanne). 2026 Jun 23;13:1816835. doi: 10.3389/fmed.2026.1816835 (PMC13337761; doi:10.3389/fmed.2026.1816835)
Supplement: Supplementary file 1 [file Data_Sheet_1.pdf]

Table S1. Feeding intolerance score[1]

Variable	Range	Points
Abdominal distension/pain	None	0
	Mild distension and no abdominal pain	1
	Moderate distension **OR** IAP 15–20 mm Hg **OR** transient abdominal pain	2
	Severe distension **OR** IAP >20 mm Hg **OR** persistent abdominal pain	5
Nausea/vomiting	None	0
	Nausea but no vomiting	1
	Nausea and vomiting without a requirement for decompression **OR** 250 ml ≤GRV <500 ml	2
	Vomiting requiring gastric decompression OR GRV ≥500 ml	5
Diarrhea	None	0
	Loose stools ≥3 times per day with 250 ml ≤volume <500 ml	1
	Loose stools ≥3 times per day with 500 ≤volume<1500 ml	2
	Loose stools ≥3 times per day with volume≥1500 ml	5

Legend: Total score = abdominal distension/pain + nausea/vomiting + diarrhea; 0–2 points: continue EN, increase or maintain initial speed, and symptomatic treatment; 3–4 points: continue EN, slow down the speed, and reevaluate EN tolerance after 2 h; ⩾5 points: suspend EN and reevaluate or replace the infusion route.

Abbreviations: IAP, intraabdominal pressure; GRV, gastric residual volume.

Table S2. Gastrointestinal Dysfunction Score (GIDS), grades of severity[2]

0 – No risk	1 – Increased risk	2 – GI dysfunction	3 – GI failure	4 – Life threatening
**No symptoms OR one of the following with oral intake**	**Two of the following**	**Three or more symptoms of score 1 OR up to two of the following**	**Three or more of the following**	**One of the following**
Absent bowel sounds<br>Vomiting<br>GRV >200 mL<br>GI paralysis/dynamic ileus<br>Abdominal distension<br>Diarrhea (not severe)<br>GI bleeding without transfusion<br>IAP 12–20 mmHg	No oral intake<br>Absent bowel sounds<br>Vomiting<br>GRV >200 mL<br>GI paralysis/dynamic ileus<br>Abdominal distension<br>Diarrhea (not severe)<br>GI bleeding without transfusion<br>IAP 12–20 mmHg	Severe diarrhea<br>GI bleeding with transfusion<br>IAP >20 mmHg	Prokinetic use<br>GI paralysis/dynamic ileus<br>Abdominal distension<br>Severe diarrhea<br>GI bleeding with transfusion<br>IAP >20 mmHg	GI bleeding leading to hemorrhagic shock<br>Mesenteric ischemia<br>Abdominal compartment syndrome

Legend: If some variables (e.g. GRV or IAP) have not been measured, the score can be calculated without considering these variables.

Abbreviations: GRV - gastric residual volume; GI – gastrointestinal; IAP - intra-abdominal pressure.

REFERENCES

1. Lin, J., et al., *Feeding intolerance score in critically ill patients with enteral nutrition: A post hoc analysis of a prospective study.* Nutr Clin Pract, 2022. **37**(4): p. 869-877.
2. Reintam Blaser, A., et al., *Development of the Gastrointestinal Dysfunction Score (GIDS) for critically ill patients - A prospective multicenter observational study (iSOFA study).* Clin Nutr, 2021. **40**(8): p. 4932-4940.
